# Influence of Long-Term Thinning on the Biomass Carbon and Soil Respiration in a Larch (*Larix gmelinii*) Forest in Northeastern China

**DOI:** 10.1155/2013/865645

**Published:** 2013-04-04

**Authors:** Huimei Wang, Wei Liu, Wenjie Wang, Yuangang Zu

**Affiliations:** ^1^Key Laboratory of Forest Plant Ecology, Ministry of Education, Northeast Forestry University, Harbin 150040, China; ^2^College of Art and Landscape, Jiangxi Agricultural University, Nanchang 330045, China

## Abstract

Thinning management is used to improve timber production, but only a few data are available on how it influences ecosystem C sink capacity. This study aims to clarify the effects of thinning on C sinks of larch plantations, the most widespread forests in Northeastern China. Both C influx from biomass production and C efflux from each soil respiration component and its temperature sensitivity were determined for scaling-up ecosystem C sink estimation: microbial composition is measured for clarifying mechanism for respiratory changes from thinning treatment. Thinning management induced 6.23 mol C m^−2^ yr^−1^ increase in biomass C, while the decrease in heterotrophic respiration (*R*_*h*_) at the thinned sites (0.9 mol C m^−2^ yr^−1^) has enhanced 14% of this biomass C increase. This decrease in *R*_*h*_ was a sum of the 42% decrease (4.1 mol C m^−2^ yr^−1^) in litter respiration and 3.2 mol C m^−2^ yr^−1^ more CO_2_ efflux from mineral soil in thinned sites compared with unthinned control. Increases in temperature, temperature sensitivity, alteration of litters, and microbial composition may be responsible for the contrary changes in *R*_*h*_ from mineral soil and litter respiration, respectively. These findings manifested that thinning management of larch plantations could enhance biomass accumulation and decrease respiratory efflux from soil, which resulted in the effectiveness improvement in sequestrating C in forest ecosystems.

## 1. Introduction

Carbon dynamics in forest plantations have become a hot topic for forest ecological researches due to their important role in global climate change [1, 2]. In recent Kyoto Protocol negotiations, it was agreed that carbon sequestration in intensively managed plantation forests could be used to offset industrial carbon efflux [2, 3], and this was highlighted in the Marrakesh Accords. To determine the optimal management techniques for these forests, the effects of commonly used management practices (e.g., selective thinning) on ecosystem carbon sinks need to be assessed. Thinning was originally performed to obtain larger diameter and higher quality timber, and more recently, an increasing number of studies have investigated the biomass productivity. The effects of thinning on biomass carbon accumulation have varied between studies [4–7], due to differences in thinning intensity and the length of time after thinning practice was carried out [7, 8]. Estimation of the effect of thinning on biomass carbon accumulation should be surveyed and analyzed.

China is home to the world's largest plantation forests (over 62 Mha), and selective thinning is one of the main management practices used in this region. Larch forests are widely distributed around the world at a latitude of 60 degrees north, and about 4.5 Mha of larch forests is distributed in Northeastern China [9, 10]. Comparison in boreal and temperate region indicates that larch forest in Northeastern China is more productive than that of Siberian forests, boreal evergreen, and south Boreas sites and similar to that of Europe Russia forest, boreal deciduous, and temperate evergreen forests [11]. Therefore, quantification of the influence of long-term thinning on the ecosystem carbon budget in these forests is of both scientific and economic significances [3, 5, 6].

Owing to the large storage, a small change in soil carbon from soil heterotrophic respiration changes can reverse the direction of forest ecosystem balance [12]. Instead of biomass carbon alone, net ecosystem productivity (the difference between annual biomass carbon increase (NPP) and soil heterotrophic respiration (*R*_*h*_)) is often used to assess ecosystem carbon sink capacity as well as in international carbon trading [3, 13]. To calculate net ecosystem productivity, *R*_*h*_ needs to be distinguished from root autotrophic respiration, and few partitioned data is available for thinning management of forests. This makes it difficult to evaluate the effects of thinning on the ecosystem carbon sink. Given that a decrease of *R*_*h*_ after thinning practices, enhancement of forest carbon sink could be observed via decreasing CO_2_ efflux from soil. Or else, the biomass carbon gain from NPP may be offset by the increase in *R*_*h*_. A long-term respiration separating *R*_*h*_ (litter decomposition and microbial respiration in mineral soil) and autotrophic respiration from roots may facilitate the quantification of *R*_*h*_ changes owing to thinning management [12, 14, 15].

Forest management has been shown to have profound but inconsistent influences on soil respiration [16–18]. This inconsistent effect may be related to the composition of the microbial community [19, 20], thermal condition alteration [21], temperature sensitivity changes [14, 15], and as litter amount and composition. Removal trees and shrubs in thinning management may possibly alter soil respiration and its contribution to different components, and as the decomposer, the changes in soil microbes are important [21]. A full check on soil microbial composition will help the understanding of underlying mechanism of thinning effects on soil carbon processes [14, 21].

The aims of this study were (1) to quantify the effects of long-term thinning on ecosystem carbon sink capacity via survey of biomass carbon and soil respiratory efflux and (2) to check the microbial and thermal changes for soil respiratory alteration from thinning treatment.

## 2. Materials and Methods

### 2.1. Study Site and Experimental Design

The study was conducted at Laoshan Experimental Station (127°34′41′′ E, 45°20′45′′ N). The larch plantation (*L. gmelinii*) being studied was afforested in 1969 at an initial planting density of 3300 plants·ha^−1^. Thinning was performed on 3 occasions following afforestation: at 11 years (1980), 20 years (1989), and 25 years (1994). The removal of the first and third thinnings was approximately 200 tree·ha^−1^, while the second thinning was approximately 300 tree·ha^−1^. During thinning, weak larches and other competing shrubs and saplings under the canopy were removed. The unthinned site is a long-term permanent plot located in the same larch plantation. This site was established in 1978 and has no artificial thinning except dead tree movement for timber utilization. Three replicating pairs of thinned and unthinned treatments (ca. 20 m ∗ 20 m) were selected in this paper. For each replicating pair, thinned plot and unthinned plot are neighbor for securing the data reliability. Area of the thinned site (2.5 ha) and unthinned site (0.5 ha) is approximately 3 ha.

### 2.2. Soil Respiration Partitioning and Environmental Parameters

Owing to its simple and cost effective and realistic respiration partitioning [22, 23], the trenched box method was used to partition respiration by autotrophic roots from respiration by heterotrophic soil microbes and litter decomposition. Each trenched box was 50 cm × 50 cm × 50 cm deep and settled permanently during the measurements. Soil respiration was measured using a Li-6400 system (LI-COR Inc., USA), and measurements were taken at least 12 hours after PVC collars (inner diameter = 10.2 cm,  height = 5 cm) insertion to avoid soil disturbance from affecting the result. At each unthinned and thinned site, 4 trenched boxes were settled to make respiration measurements. Within each trenched box, microbial respiration in the mineral soil excluding recognizable litters and roots (*R*_*m*_) was measured, while outside the trenched box, soil respiration excluding recognizable litters (*R*_−litter_) and total soil respiration (*R*_*t*_) were measured. Root respiration can be calculated as the difference between *R*_−litter_ and *R*_*m*_, while respiration from litter decomposition is the difference between *R*_*t*_ and *R*_−litter_. Duration of the measurement was in the growing season (end of April to early Oct) from April 2005 to September 2007. The measurements were carried out one time per month.

At both the thinned and unthinned sites, a thermometer probe (Li-6400 system) was used to measure soil temperature at a depth of 5 cm at the same time as soil respiration measurements were taken. Continuous soil temperature data were also recorded at 30 min intervals using a thermo Recorder mini Rt-21s (Espec, Japan) from 2005 to 2007. These data were used to compare thermal conditions at the thinned and unthinned sites and were scaled-up to estimate whole ecosystem respiration.

### 2.3. Tree Growth and Biomass-Related Parameters

An inventory of the arbor layer species larch (*L. gmelinii*), birch (*Betula platyphylla*), and ash (*Fraxinus mandshurica*) was carried out at both the thinned and unthinned sites. Tree height (*H*) and diameter at breast height (DBH) were recorded, and tree density was calculated for each species. Similarly, measurements of the height, basal diameter, and tree density of 7 understory shrubs and saplings (*Syringa amurensis*, *F. mandshurica*, *Ulmus propinqua*, *Tilia amurensis*, *Corylus heterophylla*, *Pinus koraiensis,* and *Acer mono*) were also carried out using 4 quadrats (5 m × 5 m) at both the thinned and unthinned sites. Following the arbor layer censuses, 10 to 18 different-sized individuals of each species were harvested to determine the biomass of each of plant parts (leaves, stems, branches, and roots); no such distinction between different organs was made for the understory species. Oven-dried biomass (108°C) was used to determine the allometric relationship between biomass and DBH^2^ *H* for each species (see Table s1 in Supplementary Materials available online at http://dx.doi.org/10.1155/2013/865645). These relationships were then used to determine the difference in total biomass between the thinned and unthinned sites.

### 2.4. Soil Microbial Carbon and Microbial Composition

Soil microbial carbon was measured using the chloroform (CHCL_3_) fumigation method, which was first proposed by Jenkinson and Powlson [24] and subsequently revised by Lin et al. [25]. Six soil samples were collected from a depth of 0–10 cm from both the thinned and unthinned sites in summer (August). Enumeration of soil bacteria, fungi, and actinomycetes was carried out using a plate counting method [26]. The number of colony-forming units (CFU) was then counted, and a CFU number per unit of fresh soil was calculated.

### 2.5. Data Analysis

The relationship between soil respiration and soil temperature was expressed by the exponential relationship: *R*_*s*_ = *R*_0_ *e* ^*bT*^, where *R*_*s*_ is the measured soil respiration rate (*R*_*m*_, *R*_−litter_, and *R*_*t*_), *T* is the measured soil temperature, and *R*_0_ and *b* are the best-fitting coefficients. *R*_0_ is theoretical soil respiration rate at 0°C. *Q*_10_ was then calculated using the expression exp(10∗*b*), to assess temperature sensitivity [15]. The best-fitted equation from thinned sites and unthinned sites was used to scale-up respiration of each component (mineral soils, litters and roots) from the continuous measurement of soil temperatures.

The differences of diameter, tree height, biomass of trees between different treatments, respiration between different treatments as well as among measured seasons were statistical analyzed by SPSS 17.0.

## 3. Results

### 3.1. Impact of Long-Term Thinning on Forest Biomass Carbon

Long-term thinning had a significant effect on tree size and density and most parameters for larch were statistically significant (*P* < 0.05) (Table 1). DBH and *H* of larch trees were, on average, 3.7 cm and 0.7 m larger, respectively, at the thinned site than at the unthinned site. Similarly, DBH and *H* of ash were 3.9 cm and 2.8 m larger, respectively, at the thinned site than at the unthinned site. In contrast, DBH and *H* of birch were 2.6 cm and 0.6 m smaller at the thinned site than at the unthinned site. Three arbor species were generally present at a higher density at the unthinned site than at the thinned site, and overall, total density was 20% higher at the unthinned site (1263 individuals·ha^−1^) than at the thinned site (1050 individuals·ha^−1^). The basal diameter and height of shrubs and saplings were 0.78 cm and 0.4 m smaller, respectively, at the thinned site than at the unthinned site (Table 1).

The allometric relationships (Table s1) and inventory data were used to calculate differences in biomass accumulation between the thinned and unthinned sites (Table 1). The biomass of the arbor layer at the unthinned site (191.2 Mg·ha^−1^) was lower than that at the thinned site (231.1 Mg·ha^−1^) (*P* < 0.05), while the biomass of the shrub layer at the unthinned site (2.51 Mg·ha^−1^) was approximately 1.0 Mg·ha^−1^ higher than that at the thinned site (*P* > 0.05). By summing the biomass of the 2 layers, total biomass at the unthinned site was 38.9 Mg·ha^−1^ (approximately 20%) lower than that at the thinned site. In total, arbor biomass carbon at the thinned site was 1994 g C m^−2^ higher than that at the unthinned site (*P* < 0.05), while understory carbon at the thinned site was 50 g C m^−2^ lower than that at the unthinned site (*P* > 0.05) (Table 1). By dividing the differences between the thinned site and the unthinned site by 26 years (duration of long-term thinning), we can determine the annual changes owing to the thinning treatment, about 74.8 g C m^−2^ yr^−1^ increase in total biomass carbon (Table 1).

### 3.2. Influence of Long-Term Thinning on Microbial Carbon and Composition

**Figure 1:**
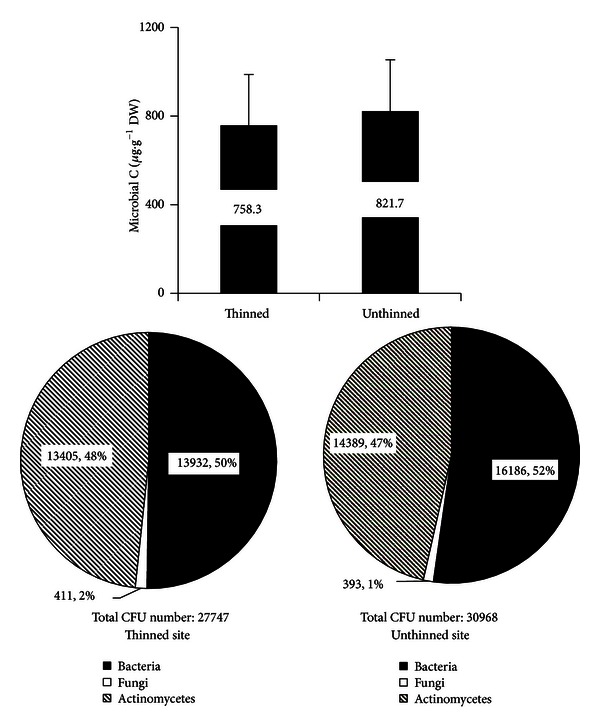
The effect of long-term thinning on microbial carbon and the microbial composition of bacteria, fungi, and actinomycetes.

Microbial carbon at the thinned site was 8% lower than that at the unthinned site (Figure 1). Similarly, total CFU number at the thinned site was 90% of that at the unthinned site (Figure 1). The main differences observed in the soil microbe composition at the thinned site were 1% increase in the percentage of actinomycetes and fungi but a 2% decrease in the percentage of bacteria (Figure 1).

### 3.3. Influence of Long-Term Thinning on Soil Temperature and Respiration Rates

**Figure 2:**
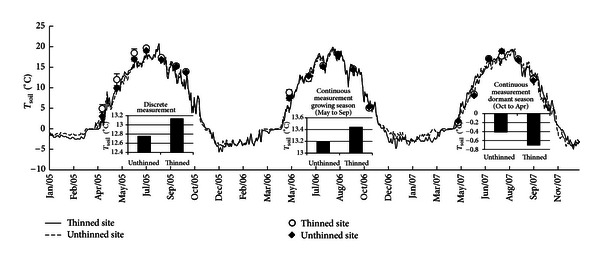
Differences in soil temperature (daily mean value) between thinned and unthinned sites. Line data were the daily mean values of continuously recorded temperatures measured using an RT21s thermometer, while the scattered data were discrete measurements that were taken at the same time as soil respiration was measured.

The effect of long-term thinning on soil temperature can be observed by comparing the continuous measurements and discrete measurements taken at the thinned and unthinned sites (Figure 2). On average across the 3-year period, the discrete soil temperature measurements were 0.38°C higher at the thinned site (13.14°C) than at the unthinned site (12.76°C). In the growing season (May to Oct) of 2005 and 2007, the continuous measurement data also showed 0.25°C higher soil temperature at the thinned site, while in the nongrowing season, contrary tendency was observed (−0.42°C  at the unthinned site while −0.71°C  at the thinned site) (Figure 2).

**Figure 3:**
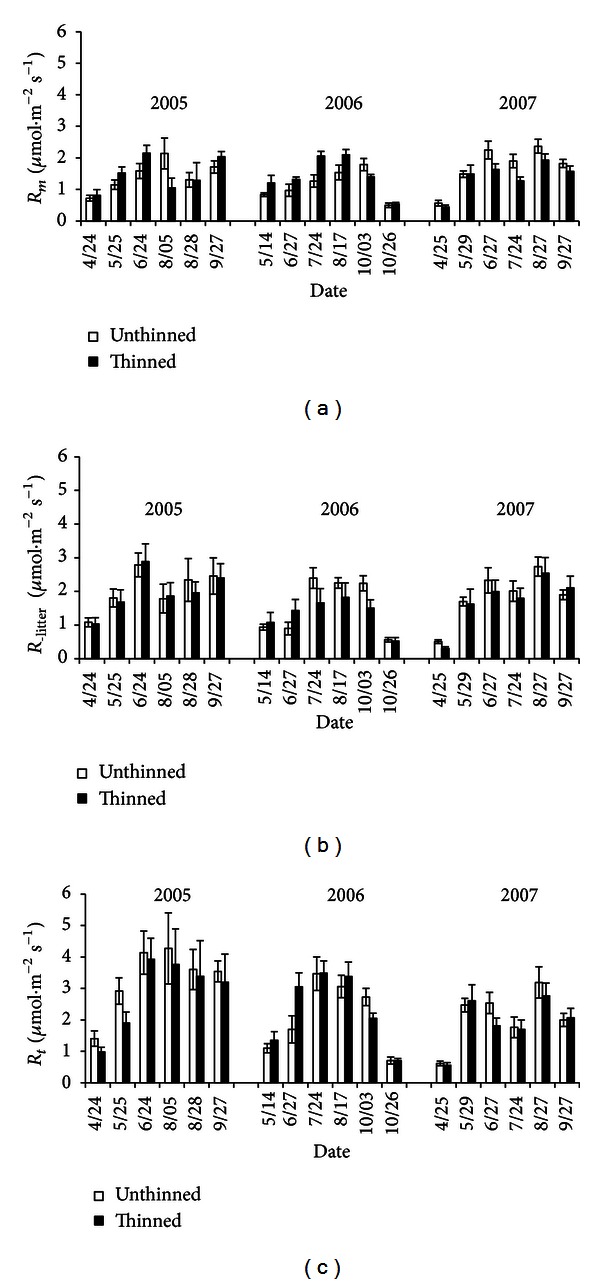
Comparison of *R*_*m*_, *R*_−litter_, and *R*_*t*_ between the unthinned and thinned sites from 2005 to 2007. (a) *R*_*m*_, (b) *R*_−litter_, and (c) *R*_*t*_.

Thinning had a varied effect on the different soil respiration components, with no general pattern being observed (Figure 3). Nine out of the 18 measurements showed that *R*_*m*_ was higher at the thinned site than at the unthinned site, while the other half showed the opposite pattern (Figure 3(a)). For both *R*_−litter_ and *R*_*t*_, most of the measurements (11 out of 18 for each) showed that the unthinned site had higher respiration rates than the thinned site, while the remainder indicated that the unthinned site had lower or similar respiration rates compared to the thinned site (Figures 3(b) and 3(c)).

By classifying the measurements by season, it was found that the respiration of soil microbes peaked in summer. In spring and autumn, soil microbes contributed more to the total soil respiration at the thinned site (73.6% and 69.2%, resp.) than at the unthinned site (55.9% and 65.2%), while levels were similar during the summer (55.2% and 54.5%) (Table s2). This seasonal difference resulted in the *R*_*m*_ contribution being 7% higher at the thinned site (65.8%) than at the unthinned site (58.8%), on average. In contrast, root respiration was generally higher at the unthinned site than that at the thinned site throughout the year, contributing by 15.2% of total respiration compared with 9.1% at the thinned site. The 3-year average litter respiration was the same at the unthinned site and the thinned site (0.69 *μ*mol m^−2^ s^−1^). However, its contribution to total respiration was slightly higher at the unthinned site in spring and autumn but lower in summer. These resulted in a slightly higher overall average contribution at the unthinned site (26.2%) than at the thinned site (25.2%) (Table s2).

### 3.4. Influence of Long-Term Thinning on Temperature Respiration Relationships and *Q*_10_ Values

**Figure 4:**
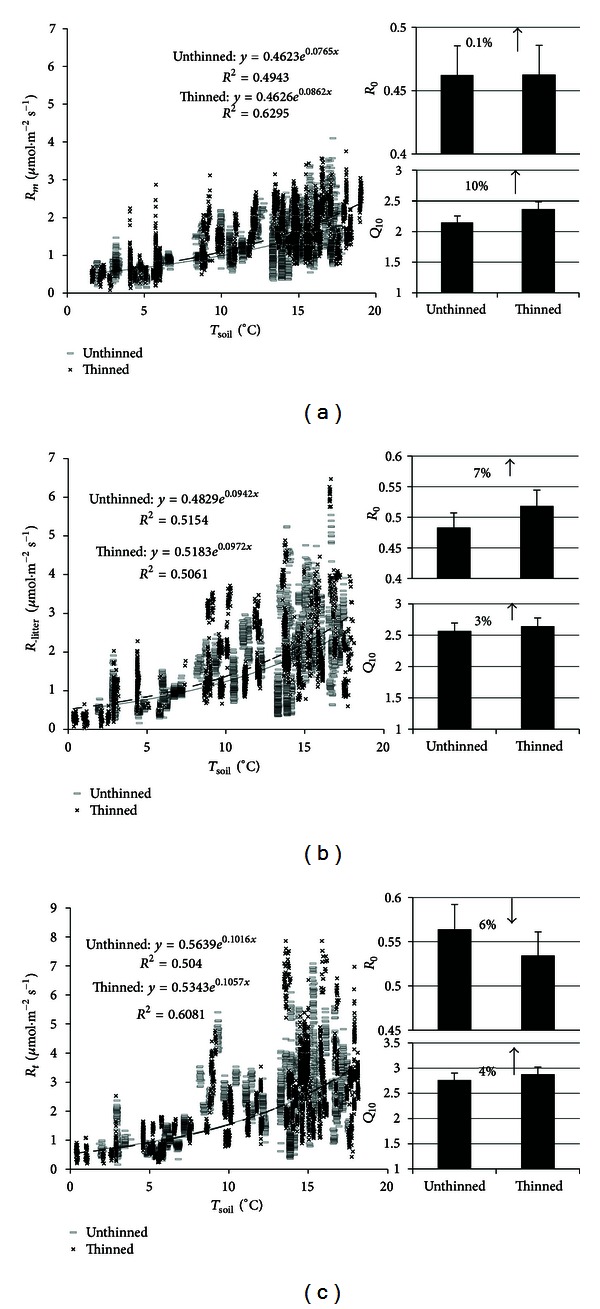
Comparison of the temporal response of *R*_*m*_, *R*_−litter_, and *R*_*t*_ between the unthinned and thinned sites from 2005 to 2007.

There were significant exponential relationships between soil temperature and *R*_*m*_, *R*_−litter_, and *R*_*t*_ (Figure 4). There was a steady increase in *b* values for *R*_*m*_, *R*_−litter_, and *R*_*t*_ at the thinned site compared with the unthinned site (Figure 4). *Q*_10_ value for *R*_*m*_ at the unthinned site (2.15) is 10% lower than that at the thinned site (2.37). Higher *Q*_10_ values for *R*_−litter_ and *R*_*t*_ at the thinned site were also observed; however, the percentage was less than 5% (Figure 4). *R*_0_ value for *R*_*m*_ was almost the same between thinned and unthinned sites. A 7% higher *R*_0_ for *R*_−litter_ but a 6% lower *R*_0_ for *R*_*t*_ at the thinned site were observed (Figure 4).

### 3.5. Influence of Long-Term Thinning on Annual CO_2_ Efflux from Soil Microbes, Roots, and Litter

Based on the continuous soil temperature data (Figure 2) and the exponential relationships (Figure 4), the respiration from soil microbes, roots, and litter was scaled-up (Figure s1). Generally, microbial respiration was higher at the thinned site, while root respiration was similar between the thinned and unthinned sites. However, respiration from litter decomposition was generally higher at the unthinned site than that at the thinned site (Figure s1).

The annual total for each component of soil respiration was calculated from Figure s1 (Table 2). Although almost no change in total respiration was found; however its distribution in different components was altered (Table 2). Heterotrophic respiration from mineral soils at the thinned site was 3.0 to 3.4 mol m^−2^ yr^−1^ higher than that at unthinned site, while respiration from litter decomposition at thinned site was 4.0 to 4.2 mol m^−2^ yr^−1^ lower than that at unthinned site. Summing of these two components of heterotrophic respiration, thinned treatment decreased 0.7–1.2 mol m^−2^ yr^−1^ (averaged at 0.9 mol m^−2^ yr^−1^) in total heterotrophic respiration. Autotrophic respiration from roots in thinned site was 0.7–0.9 mol m^−2^ yr^−1^ higher than that at the unthinned site (Table 2).

## 4. Discussion

Forest management practices such as tending and thinning can dramatically affect stand biomass and volume of harvested timber [4, 8, 27]. In this paper, average analysis showed that biomass carbon increased by 6.23 mol C m^−2^ yr^−1^ as the result of the long-term thinning (Table 1). Larch plantations are widespread in China and other northern hemisphere countries, and thinning is a common tending practice. At 2012, over 4.5 million hectares of land in Northeastern China was covered by larch forests (2 Mha in Heilongjiang Province, Liaoning Province, and Jilin Province [10] and 2.5 Mha in Daxinganling district of the Inner Mongolia autonomous region [9]). Based on our findings, approximately 12.1 Tg CO_2_ (total area 4.5 Mha × annual C sink increase, 6.23 mol m^−2^ yr^−1^ as a result of the thinning practice) could be captured annually by these managed forests compared with unthinned forests. During the same period (2005–2007), total industrial CO_2_ efflux was 670 Tg CO_2_ on average, and the annual increase was as high as 60 Tg CO_2_ in Northeastern China [28]. Thus, the management of larch forests in this region alone could offset 2% of this industrial CO_2_ efflux and 20% of its annual increase of this industrial efflux.

However, feasibility of this carbon increase used in tradeoff industrial emission depends on the ecosystem carbon sink, instead of biomass carbon alone [3, 13]. Thus, the influences on soil heterotrophic respiration should be numerated, owing to that ecosystem carbon sink equals the differences between biomass productivity and soil heterotrophic respiration. Although total CO_2_ efflux from soil was similar in the thinned site (45.4 mol m^−2^ yr^−1^) and the unthinned site (45.5 mol m^−2^ yr^−1^), the percentage of heterotrophic respiration and autotrophic respiration were altered (Table 2). On average, heterotrophic respiration of the thinning practice has decreased by 0.9 mol m^−2^ yr^−1^. This has resulted in a 14% increase in total ecosystem carbon sink at the thinned site. Therefore, calculation of ecosystem carbon sink proved that thinned treatment could enhance carbon sink size via increase in biomass and decrease in respiratory efflux. Thinning practices is a common method for managing forest [4, 27]. Its effects on biomass and soil respiration were studied previously, although conclusions are variable [2, 8, 16]. In our study, we proved that light thinning (tree density decreased from 1263 trees·ha^−1^ to 1050 trees·ha^−1^ following 3 selective thinned treatments) could be a practical silvicultural strategy to offset industrial CO_2_ emission fulfilling the requirements of the Kyoto Protocol [3, 13].

In the past, the effect of thinning on soil respiration has been found to vary. For example, investigations in a young *Pinus ponderosa* plantation demonstrated a 13% reduction in soil respiration in the first year after thinning [16]. Similar reductions have been found in other forests [1, 29]. In contrast, some other studies have found increased soil respiration in thinned stands [16, 30]. In this study, we found no overall differences in *R*_*m*_, *R*_−litter_, and *R*_*t*_ in any season using the instant measurements from 2005 to 2007 (Figures 3 and 4). Similarly, there were no differences in *R*_*t*_ between the unthinned and thinned sites when the annual data were used (Figure s1, Table 2). However, obvious alternation in its contrition in variable components was observed; that is, microbial respiration in the mineral soil at the thinned site increased, while litter respiration decreased (Table 2). The sum of these 2 components finally resulted in a decrease in total heterotrophic carbon efflux (Table 2).

These differences in respiration between the long-term thinned and unthinned sites should be related to variations in thermal condition, temperature sensitivity of respiration (*Q*_10_ values), and the soil microbial composition [12]. At the thinned site, the increased soil temperature (Figure 2) and increased temperature sensitivity of *R*_*m*_ (Figure 4) are responsible for the higher level of heterotrophic respiration from mineral soil (Table 2). On the other hand, the decrease in litter respiration at thinned site (Table 2) should be in accordance with the less return from litter both in total amount and in diversity, as shown in Table 1. Organic matter decomposition depends on specific soil microbial communities [19]. The difference in total microbial biomass decrease (biomass C and CFU data) as well as composition alteration (Figure 1) resulted in changes in the level of litter decomposition, too.

Forest thinning is one basic silviculture method to improve timber quality and productivity; this study gave a case study for the influences of thinning on ecosystem carbon sink. Just as mentioned by many previous studies, biomass C changes during forest managements are the most important item that needs to be fully considered, and our study also confirmed this. However, underground soil C dynamics should be considered in C sink evaluation, particularly in region (like northeastern China) with highly intensive agricultural exploitation [31]. In short-term instant measurement, soil respiration and its heterotrophic components showed diversified seasonal changes (Figure 3); thus instant measurement is difficult to identify the long-term effect on soil C efflux. In this study, annual sum of respiration and its components can be used to find the difference between thinning and control treatment, and data in replicating years showed similar pattern.

## 5. Conclusion

Our findings support the suggestion that long-term thinning of larch plantation in Northeastern China can improve forest carbon sink through both increase in biomass carbon and decrease in soil heterotrophic CO_2_ efflux, although the short-term instant measurement may show diversified result. Scaled-up data manifested that biomass increase could trade off 21% of the annual increase of total emission of local industry, and inclusion of soil respiration can give another 14% increase in the sink size. These findings support the use of thinning practices in larch plantation management in Northeastern China for improving ecosystem carbon sink capacity.

## Figures and Tables

**Table 1:** Long-term thinning effects on the growth and biomass C accumulation of species found in larch plantations. Values in parentheses are standard deviations.

Parameters	Treatment	Arbor layer				Shrubs and saplings	Total
		*Larix gmelinii *	*Betula platyphylla *	*Fraxinus mandshurica *	Arbor total
Diameter (cm)	Thinned	21.7 (4.7)b	19.2 (2.4)a	18.1 (9.2)a	—	1.34 (1.57)a	—
	Unthinned	18.0 (5.3)a	21.8 (3.8)a	14.2 (6.7)a	—	2.12 (2.06)a	—
Density (No·hm^−2^)*	Thinned	950 (45)a	38 (15)a	63 (16)a	1050 (40)a	10500 (550)a	—
	Unthinned	1063 (67)b	100 (18)b	100 (24)b	1263 (60)b	7700 (450)b	—
Mean height (m)	Thinned	18.5 (1.9)a	17.5 (3.4)a	16.6 (6.5)a	—	1.68 (1.22)a	—
	Unthinned	17.8 (2.0)a	18.1 (4.3)a	13.8 (6.8)a	—	2.08 (1.46)a	—
Biomass (Mg·ha^−1^)	Thinned	216.0 (12.1)a	7.1 (3.1)b	5.0 (1.3)b	231.1 (13.2)a	1.52 (0.73)a	232.6
	Unthinned	156.4 (13.1)b	15.1 (4.2)a	12.9 (3.2)a	191.2 (15.1)b	2.51 (0.63)a	193.7
Biomass C (g C m^−2^)	Thinned	10800 (605)a	355 (155)a	250 (38)b	11555 (660)a	76 (36)a	11630
	Unthinned	7820 (655)b	756 (210)b	646 (160)a	9560 (755)b	126 (31)a	9686
Difference (g C m^−2^)	Thinned-Unthinned	2980	−400	−396	1994	−50	1944
Annual C change (g C m^−2^ yr^−1^)^#^		114.6	−15.4	−15.2	76.7	−1.9	74.8

*Tree density for arbor layer species was calculated for trees of DBH > 10 cm, while for shrubs and saplings it was calculated for plants of basal diameter > 0.4 cm. ^#^The annual C change is the difference between thinned and unthinned divided by the total thinning duration (26 years).

**Table 2:** Annual effluxes from different components of soil respiration at the thinned and unthinned sites.

		Heterotrophic *R*						Autotrophic *R* from roots		Total	
Year	Items	From mineral soil		From litters		Subtotal	
		Unthinned	Thinned	Unthinned	Thinned	Unthinned	Thinned	Unthinned	Thinned	Unthinned	Thinned
2005	Amount (mol m^−2^ yr^−1^)	28.7	32.1	10.1	5.9	38.8	38.0	8.0	8.8	46.8	46.8
	Difference	3.4		−4.2		−0.8		0.8		0.0	

2006	Amount (mol m^−2^ yr^−1^)	27.7	30.7	9.7	5.5	37.4	36.2	7.6	8.3	44.9	44.6
	Differences	3.0		−4.2		−1.2		0.7		−0.3	

2007	Amount (mol m^−2^ yr^−1^)	27.7	30.9	9.6	5.6	37.3	36.5	7.5	8.4	44.8	44.8
	Differences	3.2		−4.0		−0.7		0.9		0.0	

Mean	Amount (mol m^−2^ yr^−1^)	28.0	31.2	9.8	5.7	37.8	36.9	7.7	8.5	45.5	45.4
	Differences	3.2		−4.1		−0.9		0.8		−0.1	
